# Comparison between Thermophilic and Mesophilic Membrane-Aerated Biofilm Reactors—A Modeling Study

**DOI:** 10.3390/membranes12040418

**Published:** 2022-04-12

**Authors:** Duowei Lu, Hao Bai, Baoqiang Liao

**Affiliations:** 1 Department of Chemical Engineering, Lakehead University, 955 Oliver Road, Thunder Bay, ON P7B 5E1, Canada; dlu5@lakeheadu.ca; 2 Department of Mechanical Engineering, Lakehead University, 955 Oliver Road, Thunder Bay, ON P7B 5E1, Canada; hbai@lakeheadu.ca

**Keywords:** membrane-aerated biofilm reactor, thermophilic membrane-aerated biofilm reactor, thermophilic biological treatment, biofilm, mass transfer, modeling

## Abstract

The concept of thermophilic membrane-aerated biofilm reactor (ThMABR) is studied by modeling. This concept combines the advantages and overcomes the disadvantages of conventional MABR and thermophilic aerobic biological treatment and has great potential to develop a new type of ultra-compact, highly efficient bioreactor for high-strength wastewater and waste gas treatments. Mathematical modeling was conducted to investigate the impact of temperature (mesophilic vs. thermophilic) and oxygen partial pressure on oxygen and substrate concentration profiles, membrane–biofilm interfacial oxygen concentration, oxygen penetration distance, and oxygen and substrate fluxes into biofilms. The general trend of oxygen transfer and substrate flux into biofilm between ThAnMBR and MMABR was verified by the experimental results in the literature. The results from modeling studies indicate that the ThMABR has significant advantages over the conventional mesophilic MABR in terms of improved oxygen and pollutant flux into biofilms and biodegradation rates, and an optimal biofilm thickness exists for maximum oxygen and substrate fluxes into the biofilm.

## 1. Introduction

Treatment of high-strength chemical oxygen demand (COD) industrial wastewater and waste gases has posed a significant challenge to engineers and scientists. Novel technologies for wastewater COD removal and waste gas treatment are highly desirable for sustainable development and pollution control. Recently, two promising approaches have emerged as competitive alternatives for process intensification in wastewater treatment facilities that can handle larger substrate loads and achieve higher effluent quality without increasing the footprint [1,2]. These two approaches are membrane-aerated biofilm reactor (MABR) technology [3,4] and thermophilic aerobic biological treatment (TABT) [5], which has a high oxygen transfer rate in MABR and a high biodegradation rate in TABT, and the synergy of these two technologies will develop a highly efficient and compact biological treatment system.

In an MABR system, the biofilm is immobilized on the outside of a gas-permeable membrane where the oxygen and gas pollutants are supplied for biodegradation, while the nutrients and wastewater pollutants are transported into the biofilm from the opposite direction [6]. The use of gas-permeable membranes to deliver oxygen and gas pollutants can achieve bubble-free aeration as well as extremely high removal efficiency for gas pollutants. This novel design represents a high energy efficiency compared to conventional biological treatment processes. In addition, the average TN removal in the biofilm membrane reactor was increased by around 6% compared with conventional membrane bioreactor [7]. Moreover, MABR technology is particularly suitable for the treatment of wastewaters containing volatile organic compounds (VOCs) and waste gases containing hydrophobic compounds, which are challenging to conventional aerated biological wastewater treatment and biofiltration technologies [8,9].

Nevertheless, the development of the MABR technology has been mainly a laboratory curiosity and only a few full-scale applications have been reported [3,10]. A common observation from most researchers is excessive biofilm formation (mm thickness) and decreasing pollutant flux rate with time [11]. Consequently, strategies for controlling biofilm thickness and porosity and increasing the penetration depth of oxygen, pollutants and nutrients in biofilms are crucial to improve the performance of the MABR. Unfortunately, only limited work [11] has been done in this area. It is believed that a breakthrough in biofilm structure control, particularly with respect to thickness and porosity, will lead to the development of commercial MABR technologies.

Findings from the literature review also indicate that optimization of MABR technology suffers from a lack of detailed fundamental knowledge about biofilm structure (thickness, density, porosity, diffusivity, microbial populations and their spatial distributions across biofilm depth) [12,13,14,15]. These fundamental properties have a dramatic influence on biofilm formation, transport, and reactions within biofilms. Previous studies assumed that the biofilm on gas-permeable membranes was homogeneous [16,17]. Moreover, a past work on conventional fixed biofilms suggests that there is a constantly changing population mixture and physical properties inside the biofilm [18]. Therefore, more realistic models to describe reactions and transport in biofilms will require a better understanding of biofilm structure. The full potential of MABR technology will only be realized when strategies for biofilm structure control and the relationship between biofilm structure and activity are properly understood.

The other emerging technology for waste abatement is the TABT process. It is a unique and relatively new process characterized by rapid biodegradation rates, low sludge yields, and excellent process stability [19]. Under thermophilic conditions (45–65 °C), substrate utilization rates are 3–10 times higher than those observed in mesophilic processes (25–35 °C) [20,21], and the sludge yield is similar to that of anaerobic processes [22]. These advantages have made Thermophilic MABR (ThMABR) extremely suitable for the treatment of high-strength industrial wastewater, such as pulp and paper mill effluent and food processing wastewater. However, low oxygen solubility combined with the high oxygen transfer rate required to sustain rapid biodegradation makes the selection of aeration equipment one of the most critical processes at thermophilic temperatures [23]. In addition, the poor flocculation potential and foaming problem of thermophilic bacteria represent other unique challenges for biomass separation in the suspended growth process.

In this paper, the concept of ThMABR technology is proposed and studied by theoretical analyses and modeling. Coupling the advantages of conventional MMABR technology with TABT overcomes their disadvantages and represents an innovative approach to the treatment of high-strength industrial wastewater and waste gases. On the one hand, the gas-permeable membrane is the ideal piece of aeration equipment for the delivery of the high-rate oxygen transfer required for rapid biodegradation in the ThMABR process; such rates are not achievable with conventional aeration technologies. On the other hand, the low yield and dispersing growth nature of thermophilic microorganisms represent a unique strategy for controlling the excessive growth of biofilms on the gas-permeable membrane. In addition, thermophilic treatment increases the penetration distance of oxygen, pollutants and nutrients in biofilms significantly due to increased diffusivities and decreased viscosities at thermophilic temperatures. It is anticipated that an ultra-compact, highly efficient bioreactor will be developed for high-strength wastewater and waste gas treatment through the ThMABR concept.

This communication presents theoretical analyses and modeling results of ThMABR and MMABRs. The particular interest are the differences between ThMABRs and MMABRs in terms of oxygen and pollutant flux and penetration distances and biodegradation rate.

## 2. Materials and Methods

### 2.1. Theoretical Analysis of the Impact of Temperature on Biofilm, Water and Mass Transfer Characteristics

As a biological treatment system, the ThMABR is mainly composed of membranes for oxygen delivery, and biofilms formed on membrane surfaces for biodegradation. Oxygen, pollutants and nutrients are transferred into the biofilm for biodegradation with a counter-diffusion manner. Among various factors that affect the performance of MABR, temperature plays a dominant role [24]. The various temperatures resulted in changes in biofilm characteristics (thickness, density, porosity, growth and detachment rates, microbial community, biodegradation rate, etc.), water and gas properties (viscosity, surface tension, density, etc.), membrane properties (pore size, tortuosity, solubility) and transport properties (diffusivity, flux, permeability). In return, these properties have a profound effect on the overall performance of ThMABR.

#### 2.1.1. Impact of Temperature on Biofilm Properties

**Figure 1:**
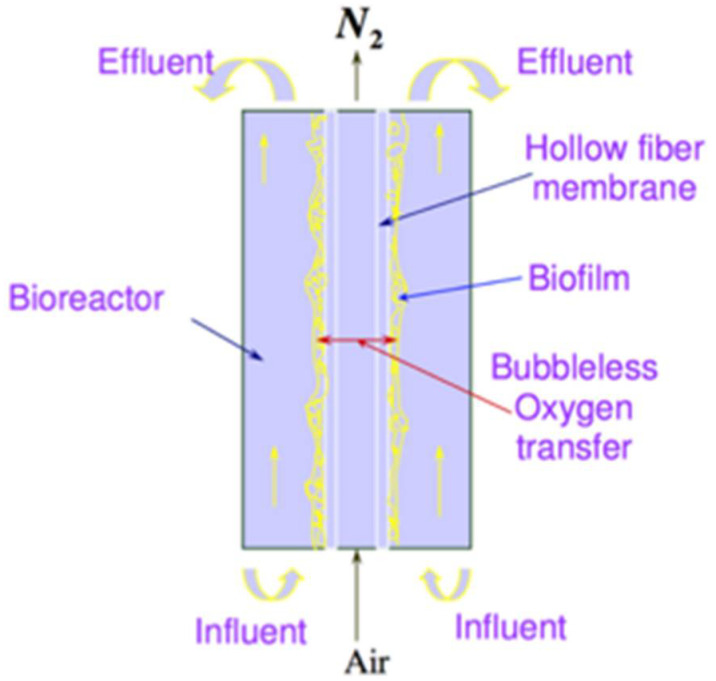
Schematics diagram of membrane-aerated biofilm reactor (MABR).

As shown in Figure 1, biofilm is the layer between the membrane surface and the bulk water phase, and mainly consists of microorganisms, extracellular polymeric substances (EPS), which are excreted by the cells, and which immobilize these cells and entrap particles within the matrix of biofilm. Biofilm is one of the most important components in MABR, as physical, chemical and biological properties of biofilms determine diffusion and biodegradation rates within the biofilm. Although extensive studies have been conducted on biofilms, the literature review indicates that most temperature-related studies focus on the formation of biofilm and very little attention has been paid to the impact of temperature on physical and chemical properties. Zhang and Bishop [25] found that the freezing technique in preparing biofilm samples for micro-slicing had no obvious adverse effects on biofilm properties (density, pore size, etc.) compared to that of the control samples. Overall, there is a lack of fundamental information on the temperature impact. However, it is clear that when the temperature is changed from the mesophilic (25–35 °C) to the thermophilic (45–65 °C) range, different microbial communities will be expected [25]. Thermophiles will survive at thermophilic temperatures and mesophiles will grow at mesophilic temperatures. 

It is generally assumed that substrate consumption rate $r_{s}$ within a biofilm can be described by Monod growth kinetics for two limiting substrates (oxygen and organic substrate) ($C_{s}$ and $C_{o}$):

(1) rs=μmax[Cs(Ks+Cs)][Co(Ko+Co)]

where $K_{s}$ is the substrate half-saturation constant and $K_{o}$ is the oxygen half-saturation constant.

In general, biodegradation rates are doubled for every 10 °C increase, in the range of 5–30 °C. A comparison of the biodegradation rates between the mesophilic and the thermophilic temperature may be difficult, owing to changes in microbial communities. However, it is generally accepted that biodegradation rates in thermophilic temperatures are much higher (3–10 times) than those in the mesophilic temperature range. Lapara and Alleman summarized the available biokinetic constants for the temperature range from 20 to 58 °C [2]. According to these figures of biokinetic constants against temperature [2], the maximum specific rate of microbial growth, maximum specific rate of substrate utilization and endogenous decay rate are strong functions of temperature. Although these data are obtained from the suspended growth biomass, it is believed, in principle, that similar trends will be observed for attached growth biomass. The oxygen transfer is a limiting factor in thermophilic treatment, due to the low oxygen solubility, high biodegradation rate, poor flocculation of biomass, and foaming issues. Thus, thermophilic treatment would negatively affect bacteria activities and may reduce process stability. Therefore, aeration must be precisely controlled to promote microbial activity and optimize organic removal and process stability.

Diffusion in biofilms is a complicated process, due to the heterogeneous nature of the biofilm structure. The pore size of channels and the porosity, tortuosity and thickness of biofilm affected the diffusivity of the oxygen and substrate. Past work assumed that the diffusivity in biofilms is equal to that in water, considering the majority of biofilm is water [23], while others consider the diffusivity in biofilm an effective diffusivity *D_eff_*, which is equal to the diffusivity in water times the physical parameters of biofilm (porosity, tortuosity, pore size) [26]. López and coworkers explained the following equation to estimate the effective diffusivity in biofilms [27].

(2) $$D_{eff}=(\varepsilon D_{w})/\tau$$

where ε is the porosity of biofilms, τ is the tortuosity factor, and $D_{w}$ is the diffusivity of water.

A change in temperature affects not only the physical properties of the bulk solution but also the physical properties of biofilms. As a result, the effective diffusivity in biofilms increases with an increase in temperature.

The impact of temperature on biofilm growth rates is generally well understood. However, very limited information is available in terms of the influence of temperature on detachment rates. It is generally believed that thermophiles have a poorer flocculating ability than mesophiles, e.g., the thermophiles have a dispersing growth nature. In addition, more substrate is converted to carbon dioxide and water instead of cell mass at thermophilic temperatures. Consequently, it is reasonable to believe that the growth rate of thermophilic biofilm thickness will be lower than that of the mesophilic biofilms under similar testing conditions.

#### 2.1.2. Impact of Temperature on Water and Gas Properties

It is well known that the physical properties of water and gas are strong functions of temperature.

Empirical equations are as follows to correlate physical properties of water and gas with temperature.

The viscosity of water equation that is accurate to within 2.5% from 0 °C to 370 °C is shown below [28]:

(3) $$\mu \left(w\right)=2.414\times 10^{-5}\times 10^{2.478/\left(T-140\right)}$$

where *T* has units of Kelvin, and μ(w) is the water viscosity which has units of Pa.s.

Sutherland’s formula can be used to derive the dynamic viscosity of an ideal gas as a function of the temperature [29].

(4) $$\mu \left(g\right)=\mu_{0}\left(T_{0}+C\right){\left(T/T_{0}\right)}^{1.5}/\left(T+C\right)$$

where μ(g) is the dynamic viscosity of gas (Pa·s or μPa·s) at input temperature *T*, $\mu_{0}$ is reference viscosity (in the same units as μ) at a reference temperature $T_{0}$, T is input temperature (K), $T_{0}$ is reference temperature (298 K), and C is Sutherland’s constant for the gaseous material in question.

Lapara and coworkers provide an excellent summary of the physical properties of water at thermophilic temperatures [30]. It is concluded that an increase in temperature from the mesophilic to the thermophilic temperature range reduces the viscosity and surface tension of water and increases mixing and colloid solubility in water, which will improve oxygen, pollutants and nutrient transfer rates. In addition, the increase in temperature reduces the saturation oxygen concentration in water and thus increases oxygen driving force across the membrane and enhances oxygen transfer.

In bulk liquid solution, diffusivities of oxygen and substrates are proportional to *T*/μ [31], that is

(5) $$\frac{D_{WT1}}{\;D_{WT2}}=\frac{T_{1}}{T_{2}}\frac{\mu T_{2}}{\mu T_{1}}$$

where $D_{w}$ is the diffusion coefficient in water, *T*_1_ and *T*_2_ are the corresponding absolute temperatures, and *μ* is the dynamic viscosity of the solvent. An increase in temperature results in a decrease in bulk liquid solution viscosity. Accordingly, diffusivities of oxygen and substrates in biofilms are proportional to *T^n^* (*n* > 1) (e.g., an increase in temperature leads to the increase in diffusivities in bulk liquid solution). The diffusivity of oxygen in the bulk liquid solution is increased from 2.1 × 10^−5^ cm^2^/s at 25 °C to 4.67 × 10^−5^ cm^2^/s at 60 °C [31].

In the lumen side of membranes, oxygen transfer to the biofilm involves adsorption, diffusion and desorption processes. According to the Chapman–Enskog kinetic theory, the diffusivity of oxygen in the bulk gas solution is proportional to *T*^1.5^/μ [31], that is

(6) $$\frac{D_{AB\left(T1\right)}}{D_{AB\left(T2\right)}}={\left(\frac{T_{1}}{T_{2}}\right)}^{1.5}\left(\frac{\mu T_{2}}{\mu T_{1}}\right)$$

An increase in temperature results in a decrease in viscosity. Consequently, the diffusivity of oxygen in the bulk gas phase is proportional to T^m^ (m > 1.5). Estimation indicates that the oxygen diffusion coefficient in water is strongly affected by temperature. This effect is even stronger in the case in air, more than doubling as temperatures increase from 20 to 60 °C [32].

#### 2.1.3. Impact of Temperature on Membrane Properties

An increase in temperature results in an increase in pore size, due to the impact of swelling [33]; thus, a high flux or permeability will be anticipated at a higher temperature. In addition, an increase in temperature leads to a lower solubility and higher diffusivity of oxygen in membranes.

Empirical correlations based on previous research data [34,35,36] are regressed using the Arrhenius equation as follows:

Oxygen solubility in Polydimethylsiloxane (PDMS) membrane:

(7) $$S_{O_{g}}=3.88014\times 10^{-11}\times e^{-58322.13/RT}$$

(Gas–PDMS membrane interface, *T* = 293–313 K)

(8) $$S_{O_{w}}=S_{O_{g}}\times H$$

(Water–PDMS membrane interface, H-Henry’s constant 0.0635, *T* = 273–333 K)

Oxygen permeability in PDMS membrane:

(9) $$P_{O_{g}}=1.1042\times 10^{-11}\times e^{-47601/RT}$$

(Gas–PDMS–Gas, *T* = 293–313 K)

Effective diffusivity of oxygen in the membrane is a function of pore diffusivity, the porosity of membrane, and the solubility of oxygen in membrane and is expressed as follows [36]:

(10) $$D_{eff}=\frac{D_{AB}\varepsilon }{\varepsilon +\left(1-\varepsilon \right)S_{Og}}$$

Temperature is an important factor that has significant degradative effects on membrane filtration because of the nature of seasonal changes in the temperature of raw water.

### 2.2. Mathematical Modelling of the Impact of Temperature on the Performance of MABR

Based on theoretical analyses and the fundamental equations that correlate the temperature and parameters mentioned above, a counter-diffusion and reaction mathematical model was developed, with the temperature impact incorporated, to study the transport and reaction processes in ThMABRs. Of particular interest is the comparison of the performance between MMABR and ThMABR.

The following set of equations was developed and used for cylindrical hollow fiber membranes.

Oxygen flux to bulk water solution without biofilms on membrane surface [37]:

(11) $$J=\left(\frac{P_{m}*H}{L_{e}}\right)\left(\frac{32*P_{O}}{H}-C_{O}|r=r_{bf\text{-}in}\right)$$

where $P_{m}$ is the permeability of oxygen; H is Henry’s constant of oxygen; $L_{e}$ is the effective thickness of silicone membrane; and *Po* is the partial pressure of oxygen gas.

Oxygen flux cross membrane can be further expressed according to oxygen concentrations in the gas phase and in the biofilm at the membrane–biofilm interface [18]:

(12) $$J=K_{d}\left(\frac{C_{o,g}}{H}-C_{o,0}\right)$$

where $C_{o,0}$ and $C_{o,g}$ are the dissolved oxygen concentrations in the membrane and biofilm bottom (g O_2_ m^−3^), *k_d_* is the overall mass transfer coefficient of oxygen (m day^−1^), and Henry’s constant is *H*.

Under steady-state conditions, diffusion and reaction of oxygen and substrate within biofilms can be described using the following equations based on Fick’s first law and Monod equation [31,37,38]:

(13) $$D_{Oeff}\left[\frac{d^{2}C_{O}}{d_{r^{2}}}+\left(\frac{1}{r}\right)\frac{dC_{O}}{dr}\right]-\left[\frac{\mu_{m}S}{K_{S}+S}\right]\left[\frac{C_{O}}{K_{O}+C_{O}}\right]\frac{X_{bf}}{Y_{XO}}=0$$

(14) $$D_{Seff}\left[\frac{d^{2}S}{d_{r^{2}}}+\left(\frac{1}{r}\right)\frac{dS}{dr}\right]-\left[\frac{\mu_{m}S}{K_{S}+S}\right]\left[\frac{C_{O}}{K_{O}+C_{O}}\right]\frac{X_{bf}}{Y_{XS}}=0$$

where $D_{Seff}$ and $D_{Oeff}$ are the effective diffusivity of substrate and oxygen in biofilm at temperature *T*, respectively; $K_{S}$ and $K_{O}$ are the half-saturation constant of substrate and oxygen at temperature *T*, respectively; $\mu_{m}$ is the maximum specific growth rate at temperature *T*; $Y_{XS}$, and $Y_{XO}$ are the biofilm yield based on substrate utilization and oxygen consumption for biofilm growth, respectively; $X_{bf}$ is the density of biofilm.

Boundary conditions can be calculated based on mass balance [31,37,39]:

r = r_bf-in_,

(15) $$D_{Oeff}\frac{dC_{O}}{dr}|r=r_{bf\text{-}in}=-\left(\frac{P_{m}*H}{L_{e}}\right)\left(\frac{32*P_{O}}{H}-C_{O}|r=r_{bf\text{-}in}\right)$$

(16) $$D_{Seff}\frac{dS}{dr}|r=r_{bf\text{-}in}=0$$

r = r_bf-out_,

(17) $$D_{Oeff}\frac{dC_{O}}{dr}|r=r_{bf\text{-}out}=\left(\frac{D_{OW}}{L_{S}}\right)\left(C_{b}-C|r=r_{bf\text{-}out}\right)$$

(18) $$D_{Seff}\frac{dS}{dr}|r=r_{bf\text{-}out}=\left(\frac{D_{SW}}{L_{S}}\right)\left(S_{b}-S|r=r_{bf\text{-}out}\right)$$

where $D_{SW}$ is the substrate diffusivity in water, $L_{S}$ is the thickness of the stagnant layer of liquid. In order to simplify computations, the linear Finite-Difference Method is introduced. In this paper, MATLAB2021a (9.10.0.1710857) was used for data calculation and analysis.

### 2.3. Model Validation

The experiment data were collected from past literature as the input in this modeling work, as shown in Table 1. The diffusion coefficients were estimated based on Equation (5) and past literature [31,40,41,42,43]. Other kinetic parameters, such as Ko and Ks, are from literature [44,45,46].

The operation conditions and information of membrane modules in literature were shown as follows. The influent was composed by a mixture of sodium acetate solution and glucose (50% glucose COD/50% sodium acetate COD in distilled water) with 1200 mg/L COD [20]. The experimental system was sequencing batch reactor MMABR and ThMABR system operated at room temperature and 55 °C, respectively. At the beginning of each reaction cycle, each batch of MABR was manually added to 1.5 L of synthetic wastewater and the reaction time was 1 day [20]. The composition details of the nutrient feed could be found in Liao and Liss’s work [20]. The membranes of MMABR and ThAnMBR are hollow fiber silicone (Model: M60-130W-200L-FC8, 13 cm wide × 20 cm long, supplied by Nagayanagi Co., Ltd., Yashio, Japan) [20].

In order to maximize the modeling results effectively, it can be used to compare the modeling results with the experimental results and examine the overall impact of reactor design and biofilm properties and operating conditions on overall MABR performance. Therefore, the past experiment comparison work about COD removal efficiency [20] in MMABR and ThMABR could be considered a validation for the present model.

## 3. Results and Discussion

The results are organized for discussion in terms of model validation using literature data, oxygen and substrate concentration profiles, biological activity profiles, membrane–biofilm interfacial oxygen concentration, oxygen penetration distance, and oxygen and substrate fluxes into biofilms under thermophilic and mesophilic conditions.

### 3.1. Model Validation 

Liao and Liss [20] found out that MABR running at a thermophilic temperature (ThMABR) was more effective than MMABR in COD removal and biofilm thickness controlling for a synthetic high-strength organic wastewater treatment. Therefore, with the same experiment parameters as the inputs at 55 °C (biofilm thickness of MMABR is 1080 μm and biofilm thickness of ThMABR is 280 μm), the general trend of model prediction on substrate removal rates could be validated by Liao and Liss’ [20] investigation. The comparison between COD removal rate in this model and literature was shown in Table 2.

The experimental results from the literature [20] verified the general trend of the higher COD removal efficiency in the ThMABR system similar to the present model. The variation of COD removal rate in the literature [20] from MMABR and ThMABR was not as significant as that predicted by the modeling study, which shows the notable change between MMABR and ThMABR. The deviation between the modeling study and experimental results could be explained by the following reasons: (1) First, the experimental data were from a sequencing batch reactor MMABR and ThMABR study and, unfortunately, the COD profile (decrease) with respect to reaction time (in one reaction cycle) was not monitored and only the COD level at the end of the reaction (24 h) was determined and used for the COD removal rate calculations. It is very likely that the majority of COD was biodegraded and reached a flat residual COD in a shorter period of time much less than 24 h (particularly for the ThMABR), and in this case, the experimental COD removal rates could be many times higher than the one reported here and much closer to the modeling results. (2) The difference between the modeled results and experimental results could also be partially caused by the back diffusion of water vapor into the lumen side of the hollow fibers, which caused additional mass transfer resistance of oxygen to biofilm. It was noted that much more water condensate was observed from the ThMABR system, due to the higher back diffusion of water vapor at the thermophilic temperature [20]. Even with this significant difference, the general tendency in both still showed that the ThMABR provided better COD removal efficiency than that of MMABR. The more rigorous validation process is still required in future work.

As Table 2 shows, the modeling and experimental results both show that an increase in the oxygen partial pressure led to an improved COD removal efficiency. These results clearly show the advantages of the ThMABR system. The ThMABR system showed a higher substrate flux or COD removal in both the modeling and experimental results. Thermophilic biofilms were much thinner than mesophilic biofilms, which implied that operating at thermophilic temperatures might be an effective approach to controlling biofilm thickness. This explains why the ThMABR performed better than the MMABR—because a thicker biofilm in the millimeter thickness range deteriorated the performance of the MMABR. Similarly, when the oxygen pressure changed to 6 psi, the substrate flux was still higher than the flux in the MMABR system. According to the experimental results, the simulated results are reasonable. The pollutant removal efficiency of ThMABR is higher than the removal in MMABR. The experimental results from the literature [20] verified the general trend of the higher COD removal efficiency in the ThMABR system.

### 3.2. Impact of Temperature (Thermophilic vs. Mesophilic) on Oxygen and Substrate Concentration Profiles

**Figure 2:**
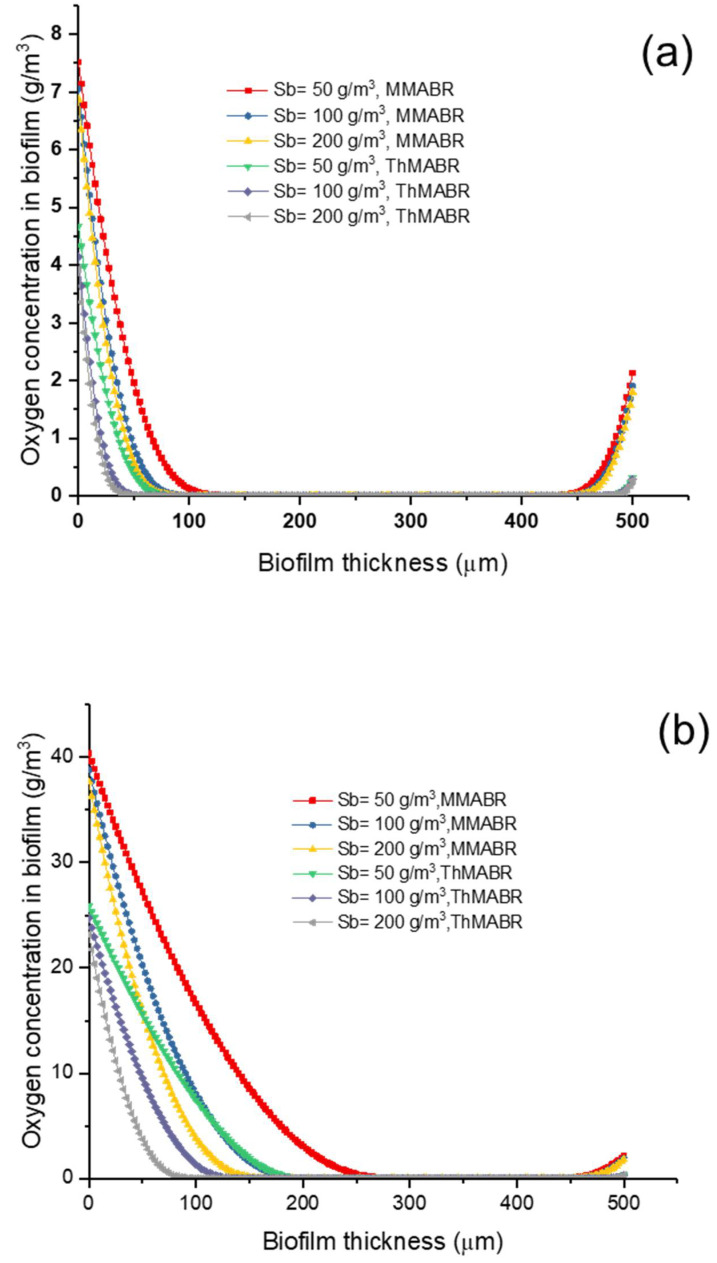
Oxygen concentration profile in MMABR and ThMABR: (**a**) air supplying; (**b**) pure oxygen supplying.

**Figure 3:**
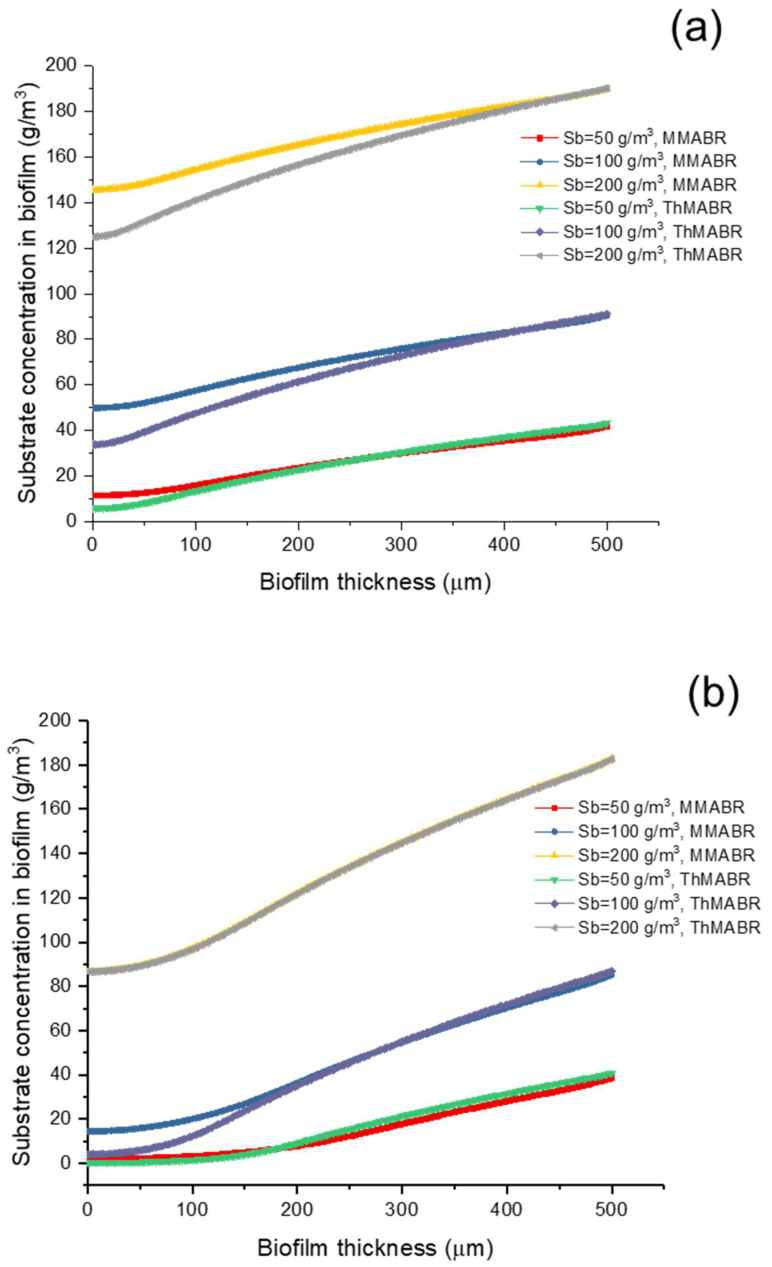
Substrate concentration profile in ThMABR and MMABR: (**a**) air supplying; (**b**) pure oxygen supplying.

Figure 2 and Figure 3 show the concentration profiles of oxygen and substrate within biofilms. The results suggest that the penetration distance of both oxygen and substrate strongly depends on the membrane–biofilm interfacial oxygen concentration. For a low substrate concentration (Sb = 50 mg/L), substrate transfer is the rate-limiting step; for a medium substrate concentration (Sb = 100 mg/L), a dual limitation (both oxygen and substrate transfer limitation) is observed in biofilms; for a high substrate concentration (Sb = 200 mg/L), oxygen transfer is the rate-limiting step. In both situations (thermophilic and mesophilic conditions), substrate either fully or partially penetrates the biofilm, while oxygen always partially penetrates the biofilms.

In most cases for municipal and industrial wastewater treatment, oxygen transfer is the rate-limiting step. Therefore, an increase in interfacial oxygen concentration is required to accommodate biological reactions in biofilms. This can be achieved by using pure oxygen for oxygen transfer. The use of pure oxygen for replacing air can increase the interfacial oxygen concentration and thus increase the penetration distance significantly [47]. Simulating the oxygen and substrate transport process in biofilm can be used to predict the pollutant removal efficiency and oxygen utilization rate. Figure 2 shows the oxygen transport process at different substrate concentrations in an MMABR and a ThMABR with air and pure oxygen supply. Compared with previous modeling studies [31,48], this profile is more reasonable, as the impact of dissolved oxygen concentration in the bulk water phase on oxygen and substrate transfer is considered here. The dissolved oxygen concentration was around 2 and 8 g/m^3^ at the end of biofilm in the bulk water phase under thermophilic and mesophilic temperatures, respectively. The oxygen profile in this simulation is similar to the result of Ntwampe et al. [49] and Matsumoto et al. [50].

In the oxygen concentration profile of the ThMABR system, the substrate concentration had a positive impact on oxygen utilization rate in both biofilm reactors. With increasing substrate concentration, the oxygen utilization rate increased. This increase stimulated the activity of microbial communities on the biofilm, which increased the reaction rate. Compared with MMABR, the oxygen concentration in the ThMABR system displayed a faster reaction rate and better oxygen utilization rate. The biofilm thickness in the ThMABR system is thinner than biofilm in the MMABR system as well. This explains why the performance of ThMABR is better than MMABR, because thicker biofilms in the millimeter thickness can degrade MMABR performance. These results also proved that the ThMABR system has more advanced points than the MMABR system. Thermophilic biofilms were much thinner than mesophilic biofilms, implying that operation at thermophilic temperatures could be an effective method to control biofilm thickness. This result is similar to Liao and Liss [20].

The substrate concentration decreased with a decreased biofilm thickness, which means a decline in the substrate utilization rate as biofilm thickness increased [43]. As shown in Figure 3a, when the substrate concentration was increased to 200 g/m^3^, a more significant difference in substrate removal could be found. A more significant substrate concentration decrease was observed in the ThMABR system. The ThMABR system had a better oxygen utilization performance, which supported that ThMABRs would provide more advanced performance on pollutant removal than the MMABR system.

If oxygen supply from the air was changed to pure oxygen, the oxygen concentration profiles under different operation conditions were totally similar. The modeling results of ThMABR still showed its outstanding removal abilities, especially for high-strength wastewater (Figure 3b). These results show that increasing oxygen partial pressure would increase reactor performance. However, the present model only considered the aerobic process in the MABR system. As the anaerobic parameters are still limited in the literature, the anaerobic process requires further study.

### 3.3. Impact of Temperature on Oxygen Penetration Distance into Biofilms

For a high-strength wastewater treatment, oxygen transfer is usually the limiting rate step. Therefore, it is important to know the penetration distance of oxygen within biofilms in order to control the biofilm thickness. The penetration distance of oxygen in a ThMABR and MMABR is shown in Figure 2a,b. The penetration distance of oxygen in MMABR is larger than that in ThMABR. This is probably not surprising, as the interfacial oxygen concentration in MMABR is always higher than that in ThMABRs. In addition, the consumption rate of oxygen in ThMABRs is higher than that in MMABRs. With substrate concentration increased, the distance of oxygen penetrated into biofilm distance was reduced. As shown in Figure 2b, when the air was replaced by pure oxygen, the penetration distance of oxygen almost doubled. This phenomenon is similar to that found by Wang and coworkers [51]. The penetrated distance in MABR was still higher than the distance in ThMABR. These results also indicate the advanced oxygen utilization of the ThMABR system.

### 3.4. Impact of Temperature on Membrane–Biofilm Interfacial Oxygen Concentration

**Figure 4:**
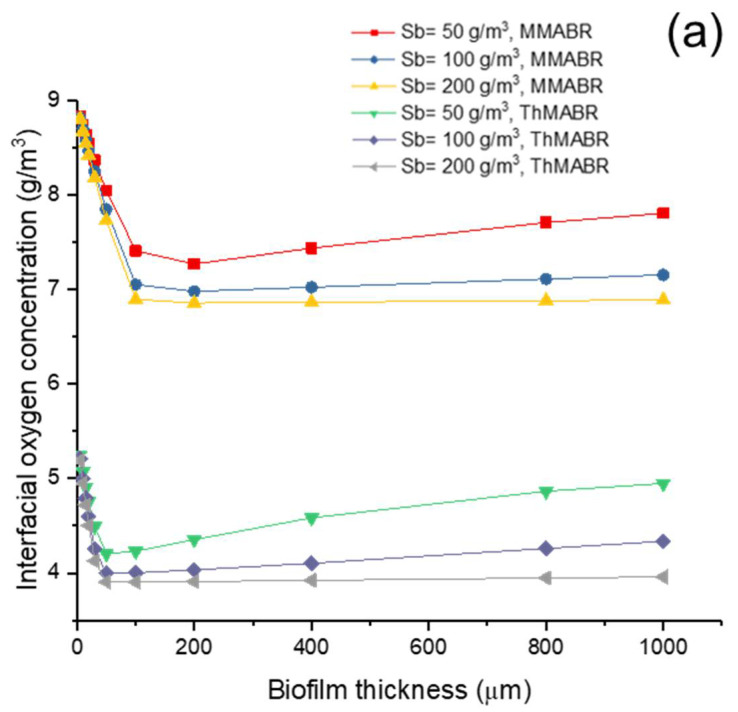

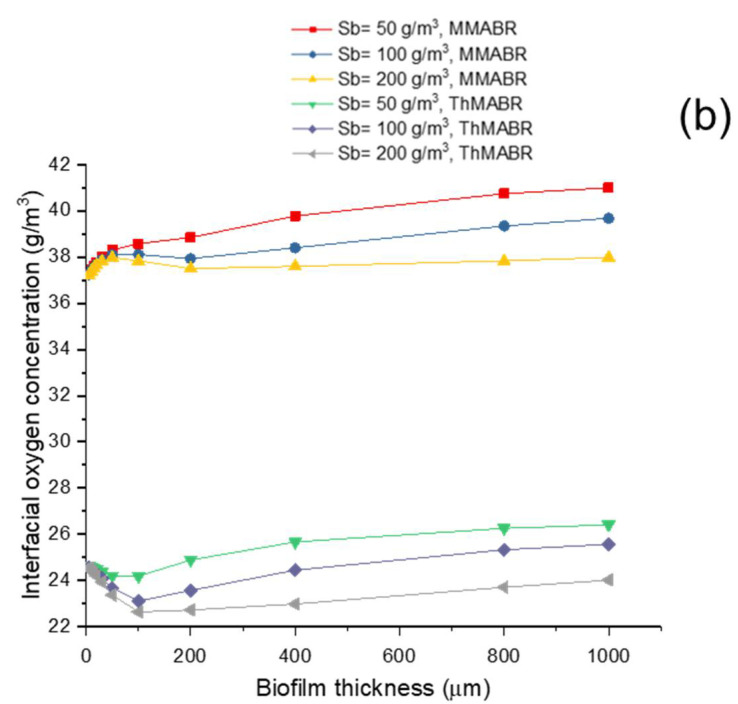
Interfacial oxygen concentration profile in ThMABR and MMABR: (**a**) air supplying; (**b**) pure oxygen supplying.

The membrane–biofilm interfacial oxygen concentration is important in determining the penetration distance of oxygen in biofilms. Usually, a high membrane–biofilm interfacial concentration is associated with a larger penetration distance of oxygen in biofilms. A comparison of interfacial oxygen concentration between ThMABR and MMABR is shown in Figure 4. The results suggest that interfacial oxygen concentration in MMABR is higher than that in ThMABR under similar conditions. Of particular interest is the presence of a minimum interfacial oxygen concentration in terms of biofilm thickness. The presence of the minimum interfacial oxygen concentration may suggest the presence of an optimal biofilm thickness for maximum oxygen fluxes into biofilms. When the biofilm thickness is thinner than the optimal biofilm thickness, an increase in biofilm thickness results in increased consumption of oxygen and thus reduces the interfacial oxygen concentration. When the biofilm thickness is thicker than the optimal biofilm thickness, a further increase in biofilm thickness introduces more transport resistance for both oxygen and substrate and thus reduces the availability of substrate concentration at the membrane–biofilm interface, which corresponds to an increase in interfacial oxygen concentration. When the biofilm thickness is thinner than the optimal biofilm thickness, an increase in biofilm thickness results in increased consumption of oxygen and thus reduces the interfacial oxygen concentration. On the other hand, when the biofilm thickness is thicker than the optimal biofilm thickness, a further increase in biofilm thickness introduces more transport resistance for both oxygen and substrate and thus reduces the availability of substrate concentration at the membrane–biofilm interface, which corresponds to an increase in interfacial oxygen concentration. An optimization point of biofilm thickness can be observed in this paper. The profile of interfacial oxygen concentration in both biofilm reactors had the lowest point at certain biofilm thicknesses, which means the highest oxygen flux could be obtained at an optimal biofilm thickness. It provided a new design idea for future lab-scale research.

Figure 4b shows that the use of pure oxygen for replacing air can increase the interfacial oxygen concentration from about 6.5–8 to 36–38 g/m^3^ in the MMABR system but only from 3.75–5.3 to 22–25 g/m^3^ for ThMABR. Thus, the pure oxygen increased the penetration distance significantly. The use of sealed hollow fibers to deliver oxygen can achieve 100% utilization of oxygen. The optimal biofilm thickness in MMABR is hard to observe. However, the optimal thickness in ThMABR increased to double. It indicated that using pure oxygen to operate the ThMABR system needs thicker thickness.

### 3.5. Impact of Temperature on Oxygen and Substrate Fluxes into Biofilms

**Figure 5:**
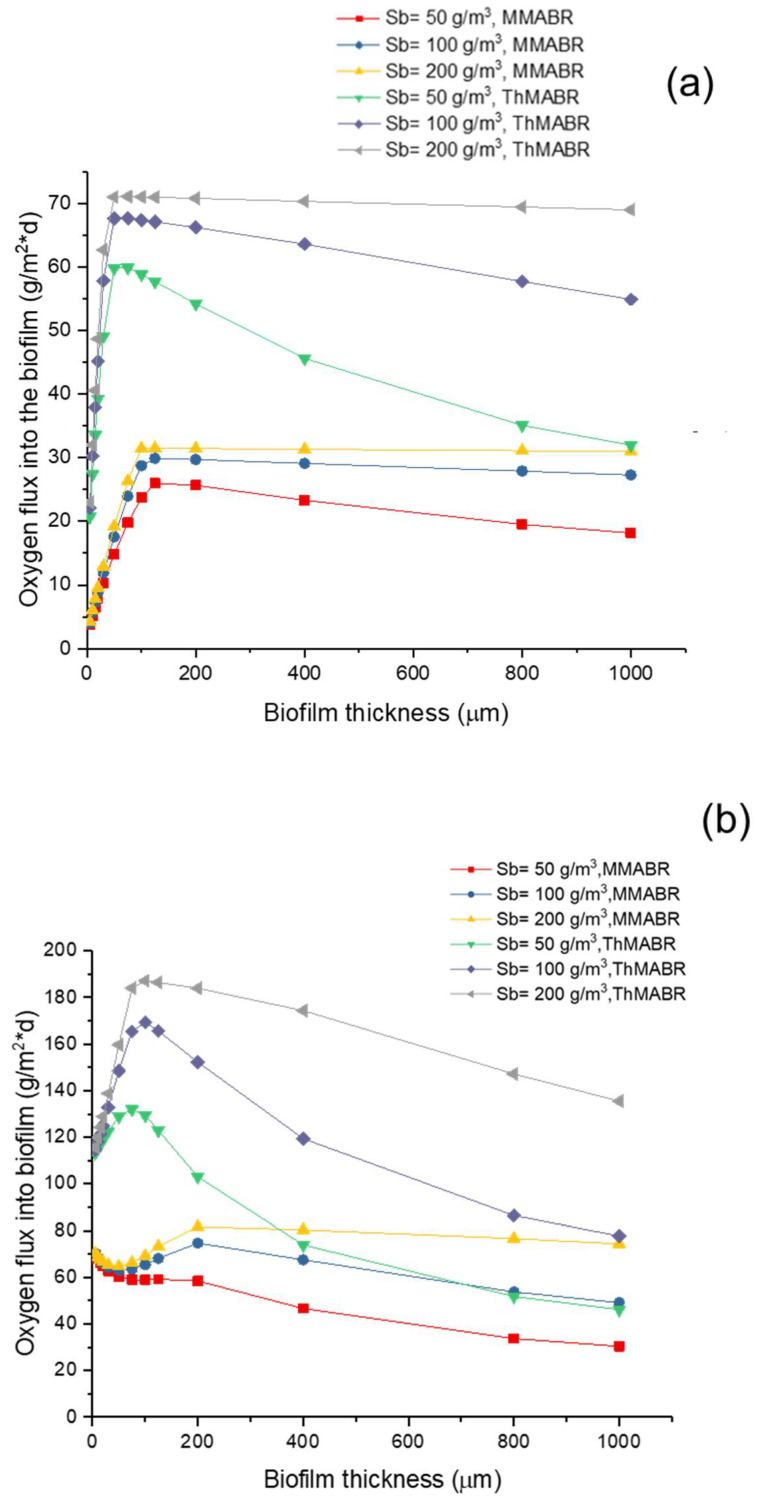
Oxygen flux comparison at different substrate concentrations: (**a**) air supplying; (**b**) pure oxygen supplying.

**Figure 6:**
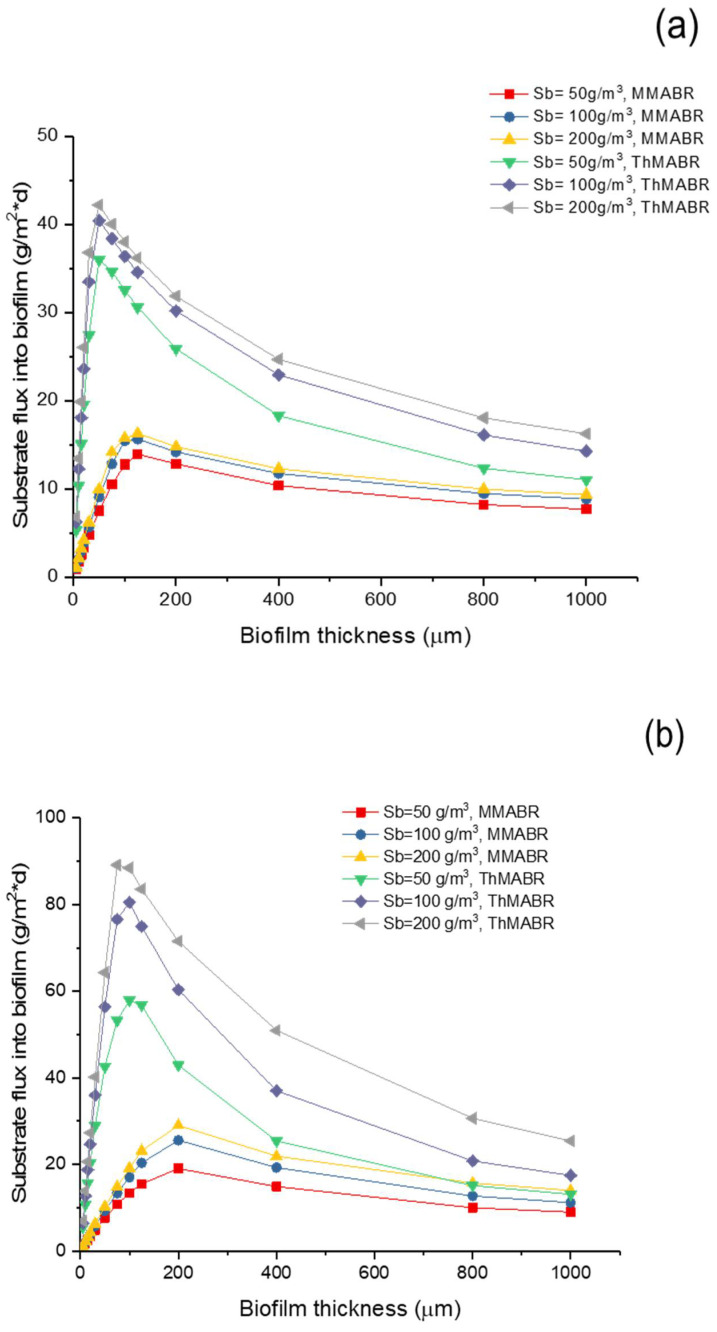
Substrate flux comparison at different substrate concentrations: (**a**) air supplying; (**b**) pure oxygen supplying.

Figure 5 and Figure 6 show the oxygen and substrate fluxes into biofilm in MMABR and ThMABR, respectively. The results suggest that the presence of a thin layer of biofilm could enhance the flux of oxygen into biofilms. This can be explained by the fact that the presence of a thin layer of biofilm would consume oxygen and thus reduce interfacial oxygen concentration, which led to an increase in oxygen flux into a biofilm. However, a further increase in biofilm thickness resulted in a minimum interfacial oxygen concentration, which corresponded to a maximum oxygen flux into a biofilm. The result indicates that an optimal biofilm thickness exists for a maximum oxygen flux into biofilms. After the optimal biofilm thickness, any further increase in biofilm thickness will introduce excessive transport resistance for oxygen and substrate transport and thus reduce the oxygen and substrate fluxes into biofilms. The optimal biofilm thickness strongly depends on the intracellular oxygen pressure [52].

A comparison of oxygen and substrate fluxes into biofilms between ThMABRs and MMABRs indicates that ThMABRs have advantages over MMABRs in terms of oxygen and substrate fluxes into biofilms. In a biofilm thickness close to the range of optimal biofilm thickness, the oxygen and substrate fluxes into biofilms in ThMABRs are about 30% higher than those in MMABRs. However, the advantages of fluxes in ThMABRs are reduced when biofilm thickness is further increased. The advantages of fluxes in ThMABRs totally disappear if the biofilm thickness is large enough. These results suggest that precise control of biofilm thickness at the range of optimal biofilm thickness is essential for achieving the advantages of ThMABRs.

According to Figure 5b and Figure 6b, the pure oxygen increased the peak of oxygen flux, which improved substrate fluxes as well. Thus, by increasing the oxygen pressure inside the membranes, we can further increase the flux of oxygen and the substrate removal rate [53]. The peak of the high-strength (Sb = 200 g/m^3^) oxygen flux decreased non-significantly in ThMABRs. It also showed thinner biofilm thickness more obviously. In both operation conditions (air and pure oxygen supplying), ThMABRs always displayed advanced removal abilities for the pollutant, which have already been applied in full-scale water treatment by their advantages. The thermophilic membrane biofilm system plants have been successfully used for pulp and papermaking wastewater treatment and food processing wastewater treatment. Both systems prove that there are many advantages compared to mesophilic bacteria. Compared to mesophilic bacteria, the biological properties of ThMABRs may be better, comparable or worse. The use of TABTs for high-temperature industrial wastewater treatment and sludge digestion significantly saves energy and enables energy-neutral or actively processed plants [54].

### 3.6. Limitations of the Present Study

Based on the modeling results discussed above, it is evident that the biofilm may have aerobic, anoxic, and anaerobic zones co-existing on the membrane surface. The current model used in this study only considered the aerobic process for COD removal and has ignored the anoxic and anaerobic processes for COD and nutrient removal. Thus, the current models are more applicable for high-strength COD industrial wastewater treatments with the need of adding nutrients based on the biological reaction stoichiometry. For more comprehensive models that account for the contributions of anaerobic COD and nutrient removals, nitrifications should be developed and integrated into the current models for a comprehensive modeling and prediction of the MMABR and ThMABR processes in the future.

The current experimental results [20] only partially validate the general trend of the modeling results in terms of COD removal rates between the MMABR and ThMABR processes. The single hollow fiber MMABR and ThMABR system and experiments should be designed to precisely validate the modeling results, such as oxygen and substrate profiles among biofilm thickness. In this case, oxygen and substrate microsensors and the biofilm thickness monitoring technique are needed to get the needed information to validate the modeling results. This should be conducted in future studies.

## 4. Conclusions

The concept of ThMABR was proposed for high-strength wastewater and gas treatments. Theoretical analyses and modeling were conducted to elucidate the advantages and disadvantages of the ThMABR, as compared to the MMABR. The main conclusions are drawn below:

(1) An increase in temperature from the mesophilic to the thermophilic range results in a significant increase in the oxygen and substrate fluxes into biofilms. The oxygen and substrate flux into biofilms at 60 °C is 2–3 times higher than that at 25 °C, respectively.
(2) Under similar operating conditions, the oxygen penetration distance of ThMABRs is smaller than that of the MMABRs, implying that the control of biofilm thickness in ThMABRs is even more important than in MMABRs.
(3) Under similar operating conditions, the membrane–biofilm interfacial oxygen concentration in ThMABR is lower than that in MMABRs.
(4) An increase in oxygen partial pressure demonstrates that the advantages of the ThMABR are even superior to that of the MMABRs in treating high-strength wastewaters.
(5) The general trend of the higher substrate removal rates observed in the modeling study of the ThMABR was partially verified by the literature experimental results, although they were not perfect. Well-controlled single-fiber MABR experiments should be designed together with biofilm microsensor techniques to verify the modeling results in the future.

## Figures and Tables

**Table 1:** Parameters for numerical modeling of diffusion and reaction in membrane-attached biofilm, MMABR and ThMABR.

Parameters	Symbol	Unit	Typical Value MMABR (25 °C)	Typical Value ThMABR (60 °C)	Typical Value ThMABR (55 °C)
Oxygen diffusivity in biofilm	*D_oeff_*	m^2^/s	$1.67\times 10^{-9}$ [41]	$3.37701\times 10^{-9}$ (Equation (5))	$3.32632\times 10^{-9}$ (Equation (5))
Substrate diffusivity in biofilm	*D_seff_*	m^2^/s	$1\times 10^{-9}$ [42]	$2.00216\times 10^{-9}$ (Equation (5))	$1.99181\times 10^{-9}$ (Equation (5))
Oxygen half-saturation constant	*K_O_*	g/m^3^	0.2 [44]	0.2 [44]	0.2 [44]
Substrate half-saturation constant	*K_S_*	g/m^3^	20 [44]	20 [44]	20 [44]
Maximum growth rate	$\mu_{m}$	1/s	$2.3148\times 10^{-5}$ [2]	$1.1574\times 10^{-4}$ [2]	$1.1574\times 10^{-4}$ [2]
Biomass yield based on oxygen	*Y_xo_*	/	0.2 [45]	0.2 [45]	0.2 [45]
Biomass yield based on substrate	*Y_xs_*	mg/mg substrate	0.45 [2]	0.35 [2]	0.35 [2]
Biofilm density	*X_bf_*	g/m^3^	55,000 [31]	55,000 [31]	55,000 [31]
Permeability	*P_m_*	gmole*m/(m^2^*s*pa)	$1.65\times 10^{-13}$ [45]	$2.81\times 10^{-13}$ [Equation (9)]	$2.73\times 10^{-13}$ [Equation (9)]
Effective thickness of hollow fiber membrane	*L_e_*	m	$7.52\times 10^{-5}$ [37]	$7.52\times 10^{-5}$ [37]	$7.52\times 10^{-5}$ [37]
Substrate diffusivity in water	*D_sw_*	m^2^/s	$1.26\times 10^{-9}$ [43]	$2.54792\times 10^{-9}$ (Equation (5))	$2.37\times 10^{-9}$ [Equation (5)]
oxygen diffusivity in water	*D_ow_*	m^2^/s	$2.41\times 10^{-9}$ [40]	$5.15\times 10^{-9}$ [40]	$4.76\times 10^{-9}$ [40]
Outside radius of hollow fiber membrane	*r_0_*	m	$3.18\times 10^{-4}$ [37]	$3.18\times 10^{-4}$ [37]	$3.18\times 10^{-4}$ [37]
Outside radius of biofilm	*r_b_*	m	$8.18\times 10^{-4}$ (This study)	$8.18\times 10^{-4}$ (This study)	$8.18\times 10^{-4}$ (This study)
Henry’s constant	*H*	atm*m^3^/mole	0.769 [46]	1.15761 [46]	1.09767 [46]

**Table 2:** The comparison between modeling predictions and experiment results from literature [20].

Biofilm Reactor	Outside Radius of Hollow Fiber (μm)	Inner Radius of Hollow Fiber (μm)	Biofilm Thickness (μm)	Simulate COD Removal Rate (g/d)	Experiment COD Removal Rate (g/d)	Relative Error
MMABR (air 4 psi)	320	200	1080 [20]	2.5780	1.1625 [20]	121.7%
MMABR (air 6 psi)	320	200	1080 [20]	2.6466	1.2375 [20]	113.8%
ThMABR (air 4 psi)	320	200	280 [20]	8.5929	1.6532 [20]	419.8%
ThMABR (air 6 psi)	320	200	280 [20]	9.0763	1.6826 [20]	439.4%

## Data Availability

Data are available upon reasonable request.

## Abbreviations

MMABR	mesophilic membrane-aerated biofilm reactor
ThMABR	thermophilic membrane-aerated biofilm reactor
TABT	thermophilic aerobic biological treatment
PDMS	Polydimethylsiloxane
*J*	flux (g/m^2^*d)
*K_la_*	overall mass transfer coefficient (min^−1^)
*T*	absolute temperature of liquid under testing (K)
*E*	modulus of elasticity of water at temperature T, (kNm^−2^)
*μ*	dynamic viscosity of the solvent
*ρ*	density of water at temperature T, (kg m^−3^)
*σ*	interfacial surface tension of water at temperature T, (N m^−1^)
*Po*	saturation pressure at the equilibrium position (atm).
*C_O,g_*	dissolved oxygen concentrations in the membrane (g O_2_ m^−3^)
*C_O,0_*	dissolved oxygen concentrations in the biofilm bottom (g O_2_ m^−3^)
*K_d_*	the overall mass transfer coefficient of oxygen (m day^−1^)
*Ko*	oxygen half-saturation constant (mg/L)
*Ks*	substrate half-saturation constant (mg/L)
*H*	Henry’s constant (atm*m^3^/mole)
μ(w)	viscosity of water (Pa·s)
μ(g)	viscosity of gas (Pa·s)
$S_{O_{g}}$	oxygen solubility in gas phase (g/L)
$S_{O_{w}}$	oxygen solubility in liquid phase (g/L)
$P_{O_{g}}$	oxygen permeability in PDMS membrane (Barrer)
*D_w_*	diffusion coefficient in water (m^2^/s)
*D_AB_*	diffusion coefficient in air (m^2^/s)
ε	porosity of biofilms
τ	tortuosity factor
COD	chemical oxygen demand
μ *_m_*	maximum growth rate (1/s)
*Yxo*	biomass yield based on oxygen
*Yxs*	biomass yield based on substrate
*Xbf*	biofilm density (g/m^3^)
*P_m_*	Permeability of oxygen gas (gmole*m/(m^2^*s*Pa)
*Le*	effective thickness of hollow fiber membrane (m)
*Ls*	stagnant layer of liquid (m)
*Dsw*	substrate diffusivity in water (m^2^/s)
*Dow*	oxygen diffusivity in water (m^2^/s)
*r_bf-in_*	outside radius of hollow fiber membrane (m)
*r_bf-out_*	outside radius of biofilm (m)
